# Nonreciprocal responses from non-centrosymmetric quantum materials

**DOI:** 10.1038/s41467-018-05759-4

**Published:** 2018-09-14

**Authors:** Yoshinori Tokura, Naoto Nagaosa

**Affiliations:** 1 RIKEN Center for Emergent Matter Science (CEMS), Wako, Saitama 351-0198 Japan; 2 Department of Applied Physics, University of Tokyo, Tokyo, 113-8656 Japan

## Abstract

Directional transport and propagation of quantum particle and current, such as electron, photon, spin, and phonon, are known to occur in the materials system with broken inversion symmetry, as exemplified by the diode in semiconductor p–n junction and the natural optical activity in chiral materials. Such a nonreciprocal response in the quantum materials of noncentrosymmetry occurs ubiquitously when the time-reversal symmetry is further broken by applying a magnetic field or with spontaneous magnetization, such as the magnetochiral effect and the nonreciprocal magnon transport or spin current in chiral magnets. In the nonlinear regime responding to the square of current and electric field, even a more variety of nonreciprocal phenomena can show up, including the photocurrent of topological origin and the unidirectional magnetoresistance in polar/chiral semiconductors. Microscopically, these nonreciprocal responses in the quantum materials are frequently encoded by the quantum Berry phase, the toroidal moment, and the magnetoelectric monopole, thus cultivating the fertile ground of the functional topological materials. Here, we review the basic mechanisms and emergent phenomena and functions of the nonreciprocal responses in the noncentrosymmetric quantum materials.

## Abstract (web summary)

A variety of directional electronic transport phenomena can occur in materials with broken inversion-symmetry. Here, Tokura and Nagaosa review the underlying mechanisms and emergent phenomena of nonreciprocal responses in noncentrosymmetric quantum materials.

## Introduction

Chirality, which characterizes right and lift, is an important and fundamental notion in whole sciences, including physics, chemistry, and biology^1^. For example, a molecule is called chiral when its mirror image does not overlap with the original molecule. In the language of symmetry, it has no inversion symmetry $\hat I$ nor mirror symmetry $\hat M$. The inversion $\hat I$can be expressed in terms of the product of the mirror symmetry $\hat M$ and the 180° rotation *C*_2_ around the axis perpendicular to the mirror plane. Therefore, $\hat I$ and $\hat M$ are identical once *C*_2_ is assumed in free space. (Hereafter, we use $\hat I$ symmetry representing either $\hat I$ or $\hat M$ depending on the crystal symmetry.) It often determines the biological functions and is crucial for the pharmaceutical biotechnology. In addition to these issues on static structures, the dynamics of the systems is the main interest in physics. Namely, the directional responses may occur in physical systems with broken $\hat I$. In solids, the crystal structure often breaks the $\hat I$ symmetry, but they do not necessarily guarantee the directional response. To see this, one can look at the one-dimensional scattering problem with the asymmetric potential barrier *V*(*x*)(≠*V*(−*x*)). The transmission and reflection probabilities are identical, irrespective of the left-going or right-going incident wave. Namely, the directional dependence is missing here. It comes from the unitary nature of the scattering (*S*)-matrix. Furthermore, the time-reversal symmetry $\hat T$ of the Schroedinger equation imposes the condition *S* = *S*^*T*^ (*S*^*T*^ means the transpose of *S*). It is natural that the right mover and left mover are exchanged by both $\hat I$ and $\hat T$, and hence these two symmetries play essential roles in the nonreciprocal responses.

Accordingly, as shown in Box 1, there are four categories of nonreciprocal responses depending on (i) the linear and nonlinear response, and (ii) $\hat T$-unbroken and $\hat T$-broken. While the physical mechanisms of the phenomena and effects listed in this table will be discussed below in detail, we stress here that nonreciprocal responses touch the most fundamental issues of condensed matter physics such as symmetries, quantum geometrical nature of electrons, and electron correlation.

### Box 1 The classification of nonreciprocal responses in noncentrosymmetric materials according to the time-reversal symmetry and linear/nonlinear nature of the responses

The nonreciprocal responses are classified into four categories, as shown in Table depending on (i) the linear or nonlinear responses, and (ii) with time-reversal symmetry or broken time-reversal symmetry. Note that the broken inversion symmetry $\hat I$ is always assumed. The nonreciprocal linear response is described by the difference of the response function between *q* and *–q* (*q*: the wavevector of the electromagnetic field), and is subject to Onsager’s reciprocal theorem Eqs. 1 and 2 in Section “Onsager reciprocal theorem” of the main text. Therefore, the diagonal response, i.e., *α* = *β* in Eq. 2, the time-reversal symmetry $\hat T$ must be broken for the different response between *q* and *–q*. An example is the directional magnetochiral effect, where the dielectric function *ε*(*q*,*ω*,*B*) differs from *ε*(−*q*,*ω*,*B*) (Section “Directional dichroism and birefringence in magnetoelectrics”). The constraint ε(*q*,*ω*,*B*) = *ε*(−*q*,*ω*,−*B*) from Onsager’s theorem requires that *B* should be finite for the magnetochiral effect. As for the off-diagonal linear response, the rotation of the polarization of light in the opposite directions between *q* and –*q* is possible for noncentrosymmetric systems even without the broken $\hat T$, i.e., the natural circular dichroism. Once the $\hat T$-symmetry is broken, we expect the directional dependence of the propagation of excitations in general. Magnon propagation in noncentrosymmetric magnets is an example, where the *q*-linear term in the energy dispersion appears (Section “Nonreciprocal dynamics of magnons”).

On the other hand, nonlinear nonreciprocal responses are characterized by the directional *I–V* characteristics, typically realized in p–n junction. In this case, the role of the wavevector *q* is replaced by the current *I* as first suggested by Rikken^40^, which leads to the resistance *R* expressed by

B1 $$R = R_0\left[ {1 + \beta B^2 + \gamma BI} \right]$$

where the resistance depends on the direction of the current *I* (Section “Unidirectional magnetoresistance”). Here it is evident that the broken $\hat T$-symmetry due to *B* is needed for the directional resistance. Also, various nonreciprocal nonlinear optical responses are realized when both $\hat I$- and $\hat T$-symmetries are broken, e.g., in multiferroics (Section “Nonlinear optical effects in multiferroics”). However, this argument is a heuristic one, and it should be noted that the breaking of $\hat T$-symmetry is not needed in the nonreciprocal response in sharp contrast to the linear response. A classic example of the nonreciprocal nonlinear response is the rectification effect of p–n junction, and another example is the shift current in noncentrosymmetric materials, where the optical excitation creating interband transitions induces the spontaneous dc photocurrent depending on the polarity of the crystal (Section “Shift current in polar insulators”). This effect does not require the $\hat T$-symmetry breaking, and is related to the Berry phase connection of the Bloch wavefunction.

## Linear nonreciprocal responses

### Onsager reciprocal theorem

In many-body systems in thermodynamic limit, the dissipative process occurs commonly, which determines the direction of time due to its irreversible nature. However, this is distinct from the time-reversal symmetry $\left( {\hat T} \right)$ breaking due to the magnetic field *B* or the spontaneous magnetization *M*. Onsager recognized the role of $\hat T$-symmetry in the microscopic dynamics of the system appearing in the macroscopic response functions linear to the external stimuli^2,3^. It is formulated by Kubo formula for linear response function *K*_*AB*_(*q*,*ω*, *B*) corresponding to the input B and output A with the wavevector *q* and frequency *ω*, which satisfies the relation^4^

1 $$K_{AB}\left( {q,\omega ,B} \right) = \varepsilon _A\varepsilon _BK_{BA}\left( { - q,\omega , - B} \right)$$

where *ε*_*A*_ = ±1, *ε*_***B***_ = ±1 specifies the even–odd nature of the quantity *A* (*B*) for the time-reversal operation. This Onsager’s reciprocal theorem, when applied to the conductivity tensor, gives

2 $${\mathrm{\sigma }}_{\alpha \beta }\left( {q,\omega ,B} \right) = \sigma _{\beta \alpha }\left( { - q,\omega , - B} \right)$$

The magnetochiral effect described in Box 1 corresponds to the formula that σ_*αα*_(*q*, *ω*, *B*) = σ_*αα*_(*ω*) + *κBq*, which is consistent with Eq. (2). Another application of Eq. (2) is the natural circular dichroism which is expressed by σ_*αβ*_ (*q*, *ω*, *B* = 0) = *ζε*_*αβγ*_*q*_*γ*_ with *ε*_*αβγ*_ being the totally antisymmetric tensor, which describes the rotation of the light polarization in a chiral crystal. This natural circular dichroism should be distinguished from the Kerr and Faraday rotation which follows from σ_*αβ*_(*q* = 0, *ω*, *B*) = *ξε*_*αβγ*_*B*_*γ*_, and does not depend on the direction of light, i.e., *q*. Below, we discuss the linear nonreciprocal responses in more detail describing their microscopic mechanism.

### Directional dichroism and birefringence in magnetoelectrics

The light wave-vector- (**q**) dependent optical response, typically termed directional dichroism or directional birefringence, can be observed ubiquitously in materials with broken symmetries of both space-inversion $\left( {\hat I} \right)$ and time-reversal $\left( {\hat T} \right)$ and in a wide photon-energy region ranging from a microwave to hard X-ray. One of the early observations is the directional light emission (luminescence) from a chiral molecule with luminescent Eu^3+^ ion under the magnetic field reported by Rikken et al.^5^; depending on the relative direction of parallel and antiparallel light-emission **q** and the applied magnetic field **B**, i.e., **q·B** > 0 or <0, the light-emission intensity is modulated, typically up to a few % for *B* of the order of a few tesla. This case is called the magnetochiral dichroism. Magnetoelectric (ME) directional anisopropy has been first observed in Er_1.5_Y_1.5_Al_5_O_12_ with the modulation of the refractive index δn = γ**q·**(**E** × **B**)^6^. When luminescent rare-earth ions are incorporated into a polar medium, e.g., in ferroelectric BaTiO_3_ doped with Er^7^, modulation of luminescence intensity occurs depending on the case, **q·**(**P** × **B**)>0 or <0; here **P** denotes the electric polarization. When the breaking of $\hat T$ is brought about by spontaneous magnetization *M* instead of applied magnetic field *B*, even a larger directional effect can show up. The directional dichroism is phenomenologically described by the Maxwell equations plugged in with the ME susceptibility tensor **α; P** = **αB** or **M** = ^**t**^**αE**. In case of multiferroics where the spontaneous **P** and **M** coexist, the diagonal and off-diagonal components of **α** are outcomes of the free energy terms proportional to **E·B**^8^ and **E** **×** **B**^6^, respectively.

**Fig. 1:**
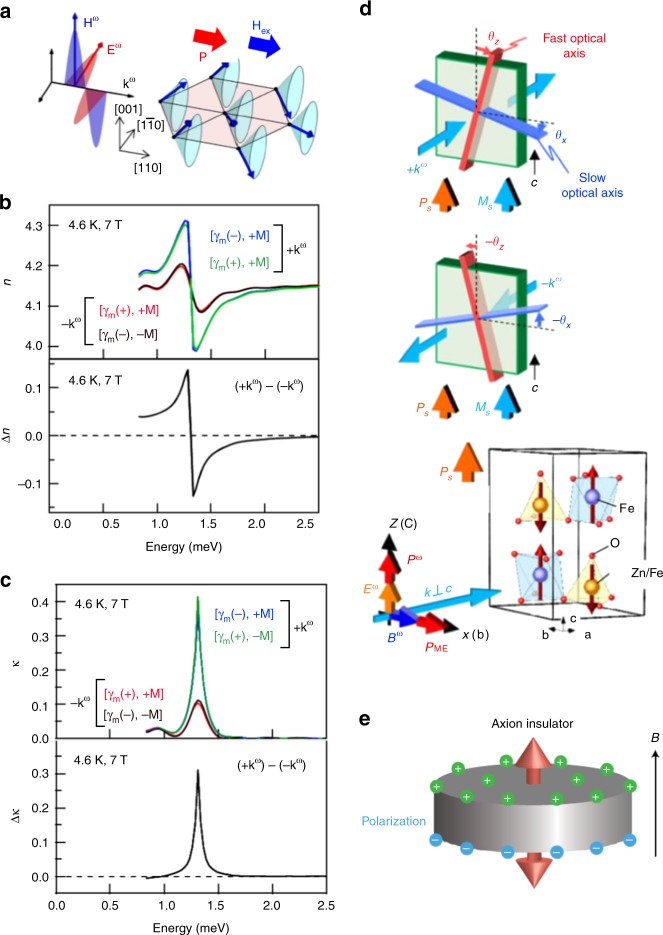
Magnetochiral dichroism and gyrotropic birefringence (GB) in multiferroics. **a** Schematic illustration of the conical screw spin structure under an external magnetic field (*H*_ex_) in multiferroic CuFe_1–*x*_Ga_*x*_O_2_ (*x* = 0.035). The *H*_ex_ parallel to the in-plane magnetic modulation *q*_m_ (e.g., // [110]) modifies the proper screw spin structure to the conical one. The ferroelectric polarization (*P*) driven by the screw spin structure also points to the in-plane *q*_m_ direction ([110]). The electromagnon spectra of (**b**) real part *n*(*ω*) and (**c**) imaginary part *κ*(*ω*) of the complex refractive index at 4.6 K and 7 T (*H*_ex_ || [110]) with the light configuration in (**a**). Spectra with both signs of spin helicity (*γ*_m_( + ) or *γ*_m_(–)) and magnetization ( +*M* or –*M*) are shown. In the respective lower panels, magnetoelectric spectra are shown defined as (**b**) Δ*n*(*ω*) = *n*( + *k*^*ω*^)–*n*(*–k*^*ω*^) and (**c**) Δ*k*(*ω*) = *κ*(+*k*^*ω*^) –*κ*(– *k*^*ω*^)^12^. **d** Schematic of GB for light with propagation vector + *k*^*ω*^ (upper) and −*k*^*ω*^ (bottom) perpendicular to the *c* axis in the case of (Fe; Zn)_2_Mo_3_O_8_; *P*_s_ and *M*_s_ stand for the spontaneous electric polarization and magnetization. *P*^*ω*^ and *P*_ME_ show the electric polarizations induced by the electric field (*E*^*ω*^) and magnetic field (*B*^*ω*^) of light, respectively. The green plate and red and blue bars denote the sample and rotating principal optical axes, respectively. Reprinted figure with permission from ref^18^. Copyright (2017) by the American Physical Society. **e** Axion insulator potentially showing the topological magnetoelectric effect and associated GB, composed of the magnetic topological insulator with the opposite magnetization directions on the top and bottom layers. Figure from ref.^21^ with permission from Nature Publishing Group

Figure 1a–d exemplifies the directional dichroism observed for the ME spin excitation, termed electromagnon, which shows both magnetic and electric transition-dipole activities^9^. The helical or conical spin order as shown in Fig. 1a can produce the electronic chirality depending on the magnetic helicity. In this particular compound, Ga-doped CuFeO_2_ with a triangular lattice of Fe^3+^ ions^10^, the hybridization of the metal (Fe^3+^) *d* state, and ligand (O^2−^) *p* state under the influence of spin–orbit interaction can produce the electric polarization **P** along the screw wavevector (**Q**) direction, as described by the *d–p* orbital hybridization model^11^. This multiferroic character can allow one to obtain the single spin-helicity domain, or equivalently the single chirality domain, via the ME cooling procedure under **B**//**E**, since the sign of *P* depends on the spin helicity in this mechanism. Thus, when the magnetic field is applied along the **Q** direction (or the **Q** is directed along the applied magnetic field direction), the magnetochiral dichroism for the **q**^ω^//**Q** (here **q**^ω^ being the light *q* vector with the frequency of *ω*, set parallel to the *z* axis) is anticipated to stem from the off-diagonal ME coefficient (α_*xy*_) since the orthogonal light electric and mangetic fields are along *x* or *y*. Figure 1b, c exemplifies the large directional dichroism spectra, i.e., the spectra of real part (*n*) and imaginary part (*κ*) of the refractive index depending on the **q**^ω^//**P** or *–***P**^12^. The change of complex refractive index is given by the off-diagonal component of the ME susceptibility, ±*α*_*ij*_(*ω*), in conjunction with the Maxwell wave equation. The α_*xy*_(*ω*) = Δ*n*(*ω*)+*i*Δ*κ*(*ω*) shows the characteristic anomalous dispersion spectrum due to the resonance of the electromagnon.

The diagonal ME susceptibility arises from the **E·B**-type coupling term in the free energy and is found in the system endowed with the ME monopoles. Here the ME monopole is defined by the magnetic structure with the inward or outward configuration of spin moment (**m**), i.e., div **m** ≠ 0^13^. A classic example of this is the antiferromagnetically ordered state in Cr_2_O_3_, in which Cr dimers formed along the *c* (*z*) direction show the up and down spin moment configuration, i.e., the ME monopole, and the linear ME effect was first predicted^14^ and detected^15^. While Cr_2_O_3_ shows both the diagonal and off-diagonal ME effect, the diagonal component leads to the specific nonreciprocal optical effect, termed gyrotropic birefringence (GB). The GB represents the rotation of the optical principle axes depending on the light incident direction, as schematized in Fig. 1d. When the light propagates along the *y* direction (**q**^*ω*^//*y*) with the light **E**^*ω*^//*z* and **H**^*ω*^//*x*, the additional *P*^*ω*^_*x*_ and *M*^*ω*^_*z*_ components are induced inside the material by the diagonal ME susceptibility of *α*_*xx*_ and α_*zz*_, respectively. Therefore, the rotation of the optical principle axes is described in terms of the dynamic ME susceptibility α_GB_(*ω*) = α_*xx*_(*ω*)–α_*zz*_(*ω*). This was first confirmed for the Cr *d–d* excitation region in the visible range^16^.

The large GB has been observed for the electromagnon in multiferroic (Fe, Zn)_2_Mo_3_O_8_ with the ferromagnetic moment *M* and the inherent electric polarization *P* both along the *c* axis (Fig. 1d)^17,18^. The ferrimagnetic spin alignment along the *c* axis also produces the ME monopoles. The electromagnon locating at 1.4 THz (5.6 meV) shows the resonant dispersion of *α*_GB_(*ω*), giving rise to the 0.02-rad rotation and 0.04-rad ellipticity of the light polarization for the 430-μm-thick crystal at 5 K. The electromagnon resonance is turned out to contribute to about 8% of the DC (*ω* = 0) linear ME susceptibility, pointing to the important role of the electromagon excitation as the ME fluctuation in multiferroics^18^.

Another important example of multiferroics to potentially show the GB is the axion insulator state constituted from the topological insulator (TI). In the TI, the Lagrangian of the electromagnetic field can contain the axion coupling term,$\left( {\frac{\alpha }{{4\pi ^2}}} \right)\theta {\mathbf{E}} \cdot {\mathbf{B}}$, in addition to the conventional Maxwell term^19,20^. Here, *α* = *e*^2^/*ħc* is the fine structure constant and *θ* is equal to π (0 or 2π) in the topological (non-topological) insulator. When the top and bottom surface-state dispersions both show mass gaps due to the exchange coupling with the opposite magnetizations (i.e., ±1 Chern number, Fig. 1e) and the Fermi level is inside these mass gaps, the compound can be an axion insulator with the topological ME effect, as characterized by the diagonal ME susceptibility *α*_*zz*_~*α*. This state can also be viewed as possessing the ME monopole like Cr_2_O_3_, and be anticipated to show the topological GB in a quantized manner^21^.

As exemplified above, the nonreciprocal dichroism/birefringence can be ubiquitously observed in a broad family of materials system with both broken $\hat I$ and $\hat T$ in a wide photon-energy region ranging from μeV (microwave)^22^ to keV (hard X-ray) region^23^. Apart from the case of the ME excitation resonance like the electromagnon^24,25^, their magnitudes usually remain at most a few % to the original absorption intensity or to the π/2 rotation for GB. A notable exception is the case of sharp resonant crystal-field *d–d* excitations in the canted antiferromagnetic phase of CuB_2_O_4_ with noncentrosymmetric space group *I42d*^26^. There, zero-phonon line of the *d–d* transition is observed to show the huge *q*-directional change of the absorption or the nearly perfect cloaking at high magnetic field, which is ascribed to the strong interference between E1 (electric-dipole) and M1 (magnetic-dipole) transitions caused by the spin–orbit coupling^26,27^.

### Nonreciprocal dynamics of magnons

When the quasiparticle with the wave vector **k** propagates along the magnetic field **B** (or magnetization **M**) in the chiral-structure compound, the relevant response function adds the additional term *A*_MCh_, which is in proportion with *σ*[sgn(**k·H**)], to the conventional term *A*_0_; here *σ* = ±1 depends on the lattice chirality, i.e., right or left handedness of the crystal. This relation generally holds for photon, magnon, and other quasiparticles which can be sensitive to the **B** or **M**. The case of photon is the magnetochiral dichroism as described in the previous section. Here we review the studies done on the magnon (spin wave) propagation.

The most archetypal example is the spin wave propagation in the chiral-lattice ferromagnet; this was recently observed in chiral magnets such as LiFe_5_O_8_^28^, Cu_2_OSeO_3_^29^, and other intermetallic compounds^30,31^. The effective Hamiltonian of the chiral magnet can be described as the sum of symmetric (*J*) and antisymmetric (*D*) exchange interactions, cubic anisotropy (*K*) term, and Zeeman term proportional to *H*. There the asymmetric exchange interaction, called Dzyaloshinskii–Moriya (DM) interaction, originates from the relativistic spin–orbit coupling in the noncentrosymmetric (here, chiral) lattice and works as a source of nonreciprocity of magnon transport. The chiral magnet generally shows the helical (screw) spin state whose magnetic periodicity is given as *aD/J* with *a* being lattice constant. The application of *H* turns the helical spin ground state to the spin-collinear (ferromagnetic) state, where the spin wave dispersion is given by

3 $$h\nu = {\mathrm{\sigma }}\left[ {{\mathrm{sgn}}\left( {{\mathbf{k}} \cdot {\mathbf{H}}} \right)} \right] \cdot 2DSV_0\left| {\mathbf{k}} \right| + C_{{\mathrm{sym}}}$$

**Fig. 2:**
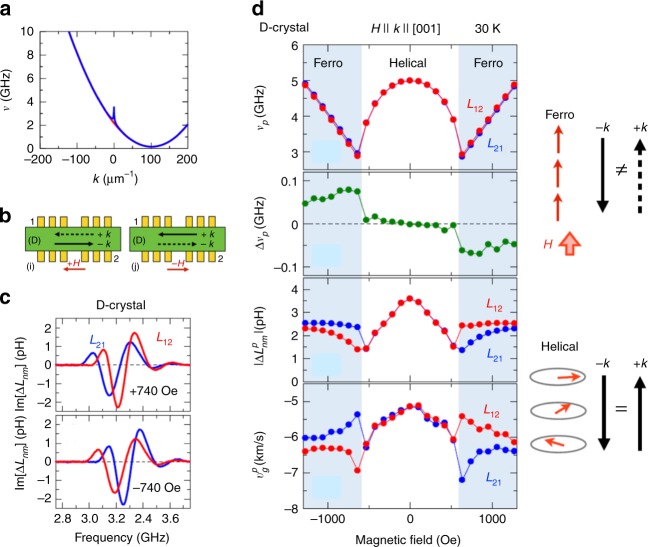
Spin wave spectroscopy on the nonreciprocal magnon transport in a chiral magnet. **a** Asymmetric spin wave dispersions for the collinear ferromagnetic state in a chiral magnet MnSi according to Eq. (3); a sharp spike around k~0 is due to the dipolar interaction. **b** The experimental setup to detect the nonreciprocity for the magnon transport between the coplanar microwave guides (ports 1 and 2). **c** Imaginary part spectra of the mutual inductance Δ*L*_12_ (Δ*L*_21_), representing the transport of the magnons from 1 to 2 (from 2 to 1) for magnetic fields applied parallel (+*H*) or antiparallel (–*H*) to the propagation direction; the result for the enantiomer D-body crystal. Another enantiomer L-body crystal shows the opposite behaviors for the exchange between Δ*L*_12_ and Δ*L*_21_. **d** Magnetic field dependence of spin wave nonreciprocity between Δ*L*_12_ and Δ*L*_21_ (i.e., +*k* and −*k*), measured for the D-body crystal of Cu_2_OSeO_3_ with the *H* // *k* // [001] configuration at 30 K. Here, *ν*_*p*_ indicates the magnetic resonance frequency giving the peak value of |Δ*Lnm*|, and Δ*ν*_*p*_ the difference between ±*k*, respectively. |Δ*L*^*p*^_*nm*_ | and *v*^*p*^_*g*_ indicate the corresponding peak value and the group velocity at the frequency *ν*_*p*_. The right panel shows the schematic illustration of collinear ferromagnetic state and helical spin state, respectively. The nonreciprocal spin wave propagation between ±*k* is discerned only in the former spin state. Reprinted figure with permission from ref^29^. Copyright (2016) by the American Physical Society

Here, *C*_sym_ = *JSV*_0_ *κ*^2^ + 2*KV*_0_*S*^3^+*γħμ*_0_ *H* is an even function of *k*; γ, *μ*_0_,*V*_0_, and *S* are gyromagnetic ratio, vacuum magnetic permeability, unit cell volume, and vector spin density, respectively. The first *k*-linear term causes the shift of the parabolic spin wave dispersion (*C*_sym_) and hence the different group velocity between +*k* and –*k*, as shown in Fig. 2a, leading to the nonreciprocal propagation of the spin wave. (In the case of the plate-shaped sample, the dispersion is modified by the magnetic dipolar interaction in the *k*~0 region, as discerned by the sharp spike around *k*~0 in Fig. 2a.)

The spin wave spectroscopy can directly demonstrate the nonreciprocal magnon transport. As the typical experimental setup (Fig. 2b), a pair of coplanar wave guides (ports 1 and 2) placed beneath the chiral magnet play roles of generator and detector of magnon. The wave number (*k*) distribution of the generated spin wave is represented by the Fourier transform of the wave guide pattern with the spacing of *λ*, i.e., *k*~2*π*/*λ*. The spectra of the mutual inductance *L*_12_ (*L*_21_) represent the propagation of the spin wave or magnon from terminal 1 to 2 (2 to 1). Figure 2c exemplifies the imaginary part of the inductance spectra *L*_12_ and *L*_21_ with *λ* ∼12 μm, taken for the specific-chirality (D-body or *σ* = +1) crystal of chiral (space group: *P*2_1_3) magnet Cu_2_OSeO_3_ with the spin-collinear state induced by the magnetic field of ±740 Oe applied parallel/antiparallel to the magnon **k** direction^29^. The magnetochiral nonreciprocal propagation can be clearly discerned as the frequency shift between *L*_12_ and *L*_21_ and also as the reversal of the shift upon the reversal of the magnetic field direction. The results for the L-body (*σ* = −1) crystal show the reversed relation, confirming the magnetochiral nature. Figure 2d summarizes the result of the spin wave spectroscopy; in the relatively high magnetic field (hatched) region where the spin-collinear ferromagnetic state is stable, the difference Δ*v*_p_ of the peak frequency of *L*_12_ and *L*_21_ (representing the magnon energy difference propagating from terminal 1-to-2 and 2-to-1) is opposite in sign in the positive and negative magnetic fields. The magnon group velocity *v*_*g*_ deduced by the *L*_12_ and *L*_21_ data differs between the 1-to-2 and 2-to-1 propagation and the difference is also reversed upon the reversal of magnetization. All these features point to the magnetochiral nonreciprocity of magnon transport, which is well accounted for with Eq. (3). By contrast, the helical spin state in the low magnetic field shows the minimal nonreciprocity although the symmetry argument would allow the nonreciprocal effect. The magnon energy difference between +*k* and –*k* is directly related to the DM interaction *D*, and in turn its observation can give the quantitative estimate of *D*, as demonstrated in a series of the chiral-lattice magnets hosting the magnetic skyrmion near room temperature^31^.

The *k*-linear term in the magnon dispersion caused by the DM interaction can be probed by other means of spin wave or magnon spectroscopy such as inelastic neutron spectroscopy^30^ and light Brillouin scattering spectroscopy^32,33^. For example, the inelastic neutron-scattering spectroscopy on chiral-lattice magnet MnSi (*P*2_1_3 space group) has directly demonstrated the asymmetric magnon dispersion as described by Eq. (3)^30^. The DM interaction produced at the interface between the heavy-element (i.e., large spin–orbit interaction) metal and ferromagnet can play an important role in spintronics function and also generate the nonreciprocal magnon transport along the lateral direction under the magnetic field applied laterally and normal to the **k** direction. This was also detected as the difference of the spin wave resonance frequency between +*k* and –*k* in terms of the light Brillouin scattering spectroscopy^32,33^.

## Nonlinear/nonreciprocal transport

### Theorem of fluctuation

Compared with the linear response theory, its nonlinear generalization of nonreciprocity is far more difficult and not well explored. However, the generalization of Onsager’s reciprocal theorem has been intensively discussed recently, and is called “fluctuation theorem”, which also originates from the time-reversal symmetry of the microscopic dynamics of the system^34–37^. Let *p*(*R*) be the probability that the entropy change is *R*. Then the fluctuation theorem claims that

4 $$\frac{{p(R,B)}}{{p( - R, - B)}} = e^{\mathrm{R}}$$

where *R* *>* 0 means the entropy production, while *R* *<* 0 the entropy reduction. *B* represents the magnetic field, and the denominator and numerator of the left-hand side of Eq. (4) are related by the time-reversal symmetry. In the thermodynamics limit, *R* becomes very large, and hence the right-hand side of Eq. (4) is infinite, and the probability of the entropy reduction is zero, i.e., the second law of thermodynamics. This theorem imposes the constraint on the fluctuation around the average, and the linear response theory as well as the Onsager’s reciprocal theorem can be derived from this theorem. Especially, the fluctuation phenomena in the mesoscopic systems are an ideal laboratory to study this theorem. As an application of the fluctuation theorem, the analysis of the counting statistics gives the relation between the nonlinear transport coefficient and noise in quantum transport of a mesoscopic conductor^38^. Let *I* be the current and *V* the voltage between the electrodes. The nonlinear *I–V* characteristics can be written as

5 $$I = G_1{\mathrm{V}} + \frac{1}{{2!}}G_2\;V^2 + \frac{1}{{3!}}G_3\;V^3 + \cdots$$

and the current-noise *S* = <(δ*I*)^2^>/Δ*f* (*δI*: the deviation of the current from the average, Δ*f*: the frequency range) is expressed as

6 $$S = S_0 + S_1\;V + \;\frac{1}{{2!}}S_2\;V^2 + \cdots$$

The fluctuation-dissipation theorem in the linear response is *S*_0_ = 4*k*_*B*_*TG*_1_, which determines the Johnson–Nyquist noise *S*_0_ in thermal equilibrium. The generalization to nonlinear response reads *S*_1_ = 2*k*_*B*_*TG*_2_. The analysis including the magnetic field *B* as well as its experimental test has been done, and the readers would refer to the literature^38,39^. Fluctuation theorems can provide the basis of the nonlinear responses beyond the Onsager’s reciprocal theorem, but their applications to the bulk transport phenomena are not well explored as yet.

### Unidirectional magnetoresistance

**Fig. 3:**
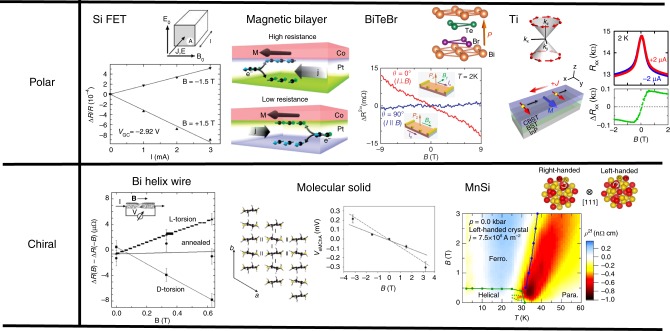
Systems showing the unidirectional magnetoresistance. There are two classes according to the crystal symmetry, i.e., polar and chiral structures. Polar systems include Si FET^41^, magnetic bilayer^42^, BiTeBr^43^, and surface state of topological insulator (TI)^44^. In this class of systems, the resistivity *R* showing the magnetochiral anisotropy has the form *R* = *R*_0_[1+*γ*(**P**×**B**)**·I**] where the directions of the polarization **P**, magnetic field **B**, and the current **I** (electric field **E**) are orthogonal to each other. There are several microscopic mechanisms of the magnetochiral anisotropy. For Si FET^41^, the relativistic Lorentz transformation and associated correction due to the factor *v/c* (*v*: velocity of electrons, *c*: velocity of light) was proposed^41^. For magnetic bilayer Ta|Co and Pt|Co systems, it has been proposed that the current-induced spin accumulation modifies the resistivity through the spin–orbit interaction and spin-dependent scattering^42^. The asymmetric band dispersion under the in-plane magnetic field in the presence of the Rashba spin–orbit interaction is identified as the origin of the nonreciprocal resistivity in BiTeBr^43^, while the asymmetric scattering of electrons by magnons is the origin in the surface state of topological insulator (TI)^44^. The examples of the other class, i.e., the chiral structure, are shown in the lower part, which shows the behavior *R* = *R*_0_[1+*γ*(**B·I**)] called the electrical magnetochiral effect. Examples of this class include Bi helix^40,46^, molecular solid^47^, and MiSi^48^. The helix structure is a representative example of this class. It has been discussed that the magnetic field **b** produced by the current is combined to the external magnetic field **B**, and the magnetoresistance for **b** **+** **B** results in the electrical magnetochiral effect in Bi helix^40^. Similar effect was also observed in a molecular semiconductor [DM-EDT-TTF]_2_ClO_4_ (middle of the lower panel)^47^. In a ferromagnet, the time-reversal symmetry is spontaneously broken, and the chiral ferromagnet can show the electrical magnetochiral effect. MnSi is a representative example, and that effect is enhanced above the helical transition temperature *T*_c_^48^. This suggests that the spin fluctuation of chiral nature is the origin of the electrical magnetochiral effect. Reprinted figures with permission from ref^40,41,45^. Copyright (2001, 2005, 2016) by the American Physical Society. Figures from ref.^42–44,47,48^ with permission from Nature Publishing Group

**Fig. 4:**
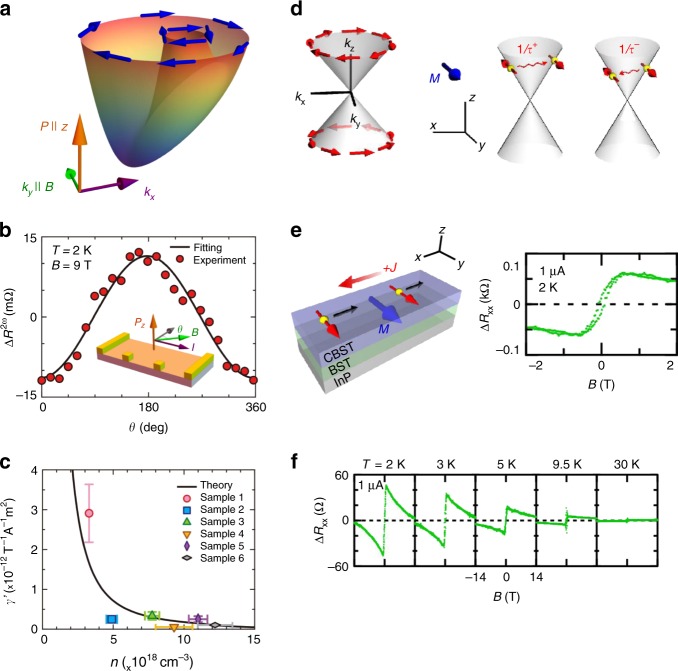
Magnetochiral anisotropy in polar systems. **a** The distorted band structure of Rashba system under the external magnetic field. When the polar axis is along the *c*-direction, the spin splitting occurs with the spin polarization for each band lying in the a–b plane. When the magnetic field is applied along *b* axis, the asymmetry between two directions along *a*-axis appears^43^. **b** The dependence of the nonlinear resistivity corresponding to the *γ* term in Eq. (B1) on the direction of the external magnetic field in BiTeBr, which is consistent with the formula *R* = *R*_0_[1+*γ*′(**P × B**)**·I**]^43^. **c** The coefficient *γ* as a function of electron density in BiTeBr. The black curve is the theoretical calculation without any fitting parameters. Figure from ref^43^. Reprinted with permission from Nature Publishing Group. **d** Schematics of the surface state of a three-dimensional topological insulator, which is described by the two-dimensional Weyl Hamiltonian with spin-momentum locking. With the in-plane magnetization along the *y*-axis, the inelastic scattering due to magnons to the right and left directions is different, leading to the nonreciprocal transport^45^. **e** The schematic experimental setup (left), and the obtained magnetic field dependence of the nonlinear resistivity^45^. **f** Magnetic field dependence of nonlinear resistivity at several temperatures. Reprinted figure with permission from ref^45^. Copyright (2016) by the American Physical Society

The directional nonlinear dc resistance has been discussed by Rikken^40^, who gave a heuristic argument generalizing the Onsager’s reciprocal theorem into the nonlinear regime, and suggested Eq. (B1) in Box 1 for the current (*I*)-dependent resistivity *R*, where the second term with coefficient *β* represents the usual magnetoresistance, while the third term with coefficient *γ* the directional resistance, i.e., magnetochiral anisotropy. Although Eq. (B1) does not describe the vector nature of the current and magnetic field, there are two types of magnetochiral anisotropy according to the crystal structures with broken inversion symmetry. One is the polar structure where the directions of the polarization **P**, magnetic field **B**, and the current **I** (electric field **E**) are orthogonal to each other; *R* = *R*_0_[1+*γ*′(**P**×**B**)**·I**]. The upper part of Fig. 3 presents the list of examples, i.e., Si FET^41^, magnetic bilayer^42^, BiTeBr^43^, and surface state of TI^42^, in this class. One typical example is the rectification effect in BiTe*X* (*X* = I, Br) in which Bi, Te, and *X* layers are stacking alternately so that the mirror symmetry along the *c* axis is broken, i.e., **P**//*c*. This material shows the giant spin splitting of the band structure due to the Rashba spin–orbit interaction^44^. However, the time-reversal symmetry leads to the relation

7 $$\varepsilon _\sigma \left( {\mathbf{k}} \right) = \varepsilon _{ - \sigma }\left( { - {\mathbf{k}}} \right)$$

with **k** being the crystal momentum and *σ* is the spin component. Therefore, when the spin components are summed up as in the case of charge transport, the directional dependence disappears. Once the external magnetic field is applied in plane, e.g., along the *y*-axis, the energy dispersion becomes asymmetric between *k*_*x*_ and *–k*_*x*_ as shown in Fig. 4a, and I–V characteristic along *a*-direction becomes nonreciprocal. Analysis in terms of the Boltzmann transport theory concludes that the coefficient γ in Eq. (B1) is independent of the lifetime of the electrons in the relaxation time approximation, similar to the case of Hall coefficient, and is an intrinsic quantity to the band structure. Therefore, one can predict quantitatively the magnitude of *γ* as a function of the electron density and temperature. Figure 4b shows the dependence of the nonlinear resistivity on the direction of the magnetic field in BiTeBr, which is consistent with the relation that *R* = *R*_0_[1+*γ*′(**P** × **B**)**·I**]^43^. Figure 4c presents the electron density dependence of *γ* measured by experiments and calculated theoretically without any fitting parameters since the band structure of this material has been already determined^43^. Excellent agreement between theory and experiment indicates that the microscopic origin of the magnetochiral anisotropy in this material is the asymmetric deformation of the energy dispersion.

Another example of the **(P × B) · I**-type nonreciprocity is the case of the TI, as shown in Fig. 4d–f ^45^. In the TI thin film (Bi, Sb)_2_Te_3_ (BST), the upper layer part (denoted CBST) is doped with magnetic Cr ions to enhance the magnetic response as well as to introduce the asymmetry between the top and bottom surface layers where the cone-like dispersion of the conduction electron is formed with strong spin-momentum locking (Fig. 4d). When the magnetic field **B** is applied along in-plane and perpendicular to the current *I* direction, the large nonlinear component Δ*R*_*xx*_ shows up (Fig. 4e); note that the spontaneous magnetization is originally (at zero field) perpendicular to the plane but inclined to the in-plane by the external in-plane field. The important feature for Δ*R*_*xx*_ is its sign reversal upon the reversal of *B* and also upon the reversal of the stacking sequence structure of BST (nonmagnetic) and CBST (magnetic) layer; the latter procedure corresponds to the reversal of *P*, thus satisfying the condition of the magnetochiral anisotropy. Under the in-plane *B* field, the Weyl cone should show a parallel shift in the *k*-space, yet this would give no effect on the nonlinear conduction if the quadratic term of the band dispersion could be neglected, being contrary to the case of the above example of the Rashba system. The unidirectional nonlinear magnetoresistance Δ*R*_*xx*_ observed in this semi-magnetic TI system shows the characteristic magnetic field and temperature dependence; as increasing *B* to several-tesla region and as elevating temperature across the ferromagnetic ordering temperature, Δ*R*_*xx*_ shows a steep reduction. These observed features are all explained in terms of the relevance of the magnon excitations, i.e., their emission and absorption, in the originally forbidden backward-scattering process of the spin-momentum-coupled Weyl electrons (Fig. 4a). The application of *B* opens the spin wave gap, resulting in the reduction of the magnon scattering events at low temperatures. Furthermore, the tuning of the Fermi level around the Dirac (band crossing) point is found to enhance Δ*R*_*xx*_ because the magnon’s **q** vector and energy to mediate the backward scattering of the conduction electron can be small and more effectively thermally populated.

The other class of the materials showing unidirectional magnetoreistance is the chiral structure shown in the lower part of Fig. 3; in this case, the nonlinear nonreciprocal conduction is sometimes called the electrical magnetochiral effect in analogy to the above-described optical magnetochiral effect. Examples of this class include Bi helix^40,46^, molecular solid^47^, and MiSi^48^. The helix is a representative example of this class, and an early experiment on Bi-helix wire found a finite *γ* value^40^. The suggested mechanism of this effect is the magnetoresistance due to the magnetic induction **b** by the current *I* circulating along the helix, i.e., the effective magnetic field felt by the electrons is **B** *+* **b**, the magnitude of which depends on the direction of *I*. In this case, the nonlinear conduction is maximized when magnetic field **B** and the current **I** (electric field *E*) are parallel to each other, namely taking the form of **B·I**. A similar effect was also observed in a molecular semiconductor^47^. Ferromagnets with chiral crystal structures are also expected to show the magnetochiral anisotropy.

Beyond the phenomenological or symmetry argument, there is known the case where a specific scattering mechanism can cause the electrical magnetochiral effect. In MnSi with the cubic chiral-lattice structure (*P*2_1_3), in which spin-helical or skyrmion phase forms below *T*_c_ = 35 K because of Dzyaloshinskii–Moriya interaction inherent to the lattice chirality, the **B·I**-type nonlinear conduction is observed with the opposite sign for the two enantiomers^48^. Symmetry allows the electrical magnetochiral effect in every *T–B* region. In reality, however, the effect becomes appreciable not in the helical magnetic order region but immediately above *T*_c_, where the spin fluctuation is observed to retain the chiral character. This means that the scattering process of the conduction electron by the chiral spin fluctuation is proven to be the major source of the electrical magnetochiral effect in this case.

### Nonreciprocal response in Weyl semimetals

Weyl semimetal is a class of materials where the Weyl fermions are at the Fermi energy typically described by the Hamiltonian^49^

8 $$H_{{\mathrm{Weyl}}} = \eta v{\mathbf{\sigma }} \cdot \left( {{\mathbf{k}} - {\mathbf{k}}_0} \right),$$

where ***σ*** = (σ^*x*^,σ^*y*^,σ^*z*^) are Pauli matrices, *v* is the velocity, **k**_0_ is the band crossing point, and *η* = ±1 specifies the chirality. It has been known that the Weyl fermion acts as the magnetic monopole or antimonopole of the emergent magnetic field in the momentum space for *η* = +1 and *η* = −1, respectively. The Nielsen–Ninomiya theorem^50^ dictates the equal number of the Weyl fermions with opposite chiralities. One of the peculiar phenomena associated with Weyl fermions is the chiral anomaly, which is shared also by the Dirac fermion with 4 × 4 Hamiltonian. This is the phenomenon where the fermions are transferred between opposite chiralities in proportion to **E·B** under the external electromagnetic field. This results in the large negative magnetoresistance when the electric and magnetic fields are parallel or antiparallel to each other^49,51^.

Note that, as for the Weyl fermions with 2 × 2 Hamiltonian, either the time-reversal $\hat T$ or the space-inversion $\hat I$ must be broken to lift the Kramer’s degeneracy except at **k**_0_. Accordingly, there are two types of Weyl semimetals. One is the $\hat T$-broken, but $\hat I$-symmetric Weyl semimetal. $\hat I$-symmetry relates the Weyl fermions at **k**_0_ and −**k**_0_, and the chiralities of these are opposite to each other. On the other hand, in the $\hat I$-symmetry broken Weyl semimetals, the Weyl fermions at **k**_0_ and −**k**_0_ have the same chirality. Therefore, according to the Nielsen–Ninomiya theorem^50^, there must be at least another pair of Weyl fermions at **k**_1_ and −**k**_1_. Namely, there are at least four Weyl fermions at ±**k**_0_ and ±**k**_1_. The energy dispersions and also the Fermi energy shift at ±**k**_0_ and ±**k**_1_ are different and contribute differently to the conductivity. Therefore, once the external magnetic and electric fields shift the electrons in proportion to **E·B** between positive and negative chirality Weyl fermions due to the chiral anomaly, there occurs the current proportional to (**E·B**)**E**, which indicates the magnetochiral anisotropy^52^. Because the Weyl fermions in $\hat I$-broken system are solely due to the spin–orbit interaction, and also the Fermi energy shift from the Weyl point is usually small, the smallness of the perturbation discussed in the previous section is avoided, leading to the large nonreciprocal response. Quantitatively, *γ* value in Eq. (B1) can be four orders of magnitude larger than those discussed in the literature^40^.

### Nonreciprocal transport in a noncentrosymmetric superconductor

The magnetochiral anisotropy is usually a small effect because it requires both the spin–orbit interaction *λ*, which reflects the inversion symmetry breaking and the magnetic field *B* breaking the time-reversal symmetry. For each perturbation, the energy denominator is typically the Fermi energy *ε*_*F*_, and *γ* is expected to be proportional to (*μ*_*B*_*B*/ε_*F*_)(*λ*/ε_*F*_) and is usually very small. In the case of BiTeBr discussed above, the giant Rashba splitting leads to *λ*/*ε*_*F*_ ∼ 1 and the reasonably large *γ*. However, even larger *γ* is realized in the noncentrosymmetric superconductors^53^ as shown below. The physical picture for this enhancement is that the Cooper pairs with the large coherence length ξ feel the noncentrosymmetric nature of the potential sensitively in the low- energy region below the superconducting gap Δ. In the *I–V* characteristics, the voltage is zero in the low current region. However, in 2D superconductors with an external magnetic field, the unpinned vortices produce the finite resistance and *γ* can be defined^54^. In this case, the energy denominator in the expression of *γ* becomes Δ replacing *ε*_*F*_, leading to the enhancement of γ by some power of the factor (*ε*_*F*_/Δ). An example is the noncentrosymmetric superconductor MoS_2_ composed of the stacked layers with weak van der Waals interaction^55^. The monolayer system is a 2D network with *D*_3h_ symmetry, leading to the trigonal-warping of the Fermi surfaces and out-of-plane spin polarization with effective Zeeman fields at *K*-points. Measurement of *γ* as a function of temperature shows its rapid growth below the temperature slightly higher than the mean field transition temperature *T*_0_, the mean field transition temperature, reaching the value *γ*~8000 T^−1^A^−1^ well below *T*_0_^56^.

Theoretical analysis has been restricted to the paraconductivity above *T*_0_^57^, concluding the enhancement of *γ* as $\frac{{\gamma _S}}{{\gamma _N}} \sim \left( {\frac{{\varepsilon _F}}{{\mathrm{\Delta }}}} \right)^3$, and predicted *γ* ~400 T^−1^A^−1^ . Although the analysis well below *T*_0_ has not yet been done, the rapid increase of γ observed experimentally is consistent with the theoretical prediction. The dynamics of vortices should be relevant to the nonreciprocal resistivity well below *T*_0_, which is left for future studies.

## Nonlinear/nonreciprocal photonic responses

### Nonlinear optical effects in multiferroics

The nonlinear and nonreciprocal optical effects are also expected generically for the materials system with broken symmetries of space-inversion and time-reversal. One important source of such nonlinear optics (NLO) in the magnetic system is the toroidal moment *T*^58^ as defined by ${\mathbf{T}} = \frac{1}{2}\mathop {\sum }\limits_i {\mathbf{r}}_i \times {\mathbf{S}}_i$; here **S**_*i*_ is the spin moment on the site **r**_*i*_. Since the effective spin–orbit coupling term in Hamiltonian is described as λ**L·S** = λ(**r** × **p**)**·S** = −*λ*(**r** × **S**)**·p** = *e***A**_eff_ **·p** where *e* and **p** are electron’s charge and momentum, the toroidal moment **T** can be viewed as the built-in vector potential **A**_eff_ under the presence of spin–orbit interaction. Then **T** can mix with the ac vector potential **A**_*ω*_ from the light (frequency *ω*, i.e., **T** **+** **A**_*ω*_). The compound may not show the second-order nonlinear optical (NLO) effect responding to $A_\omega ^2$, but generically does the third-order term responding to $A_\omega ^3$. When the *T* is mixed in, this third-order term can effectively generate the second-order response, i.e., responding to $TA_\omega ^2$. Namely, when the light electric field *E*_*ω*_ (or *A*_*ω*_) is parallel to *T* of the material, the effective second-order NLO, typically second-harmonic generation (SHG with frequency of 2*ω*) is observed. Conversely, such magnetization-induced SHG can be used as a probe for the toroidal moment.

**Fig. 5:**
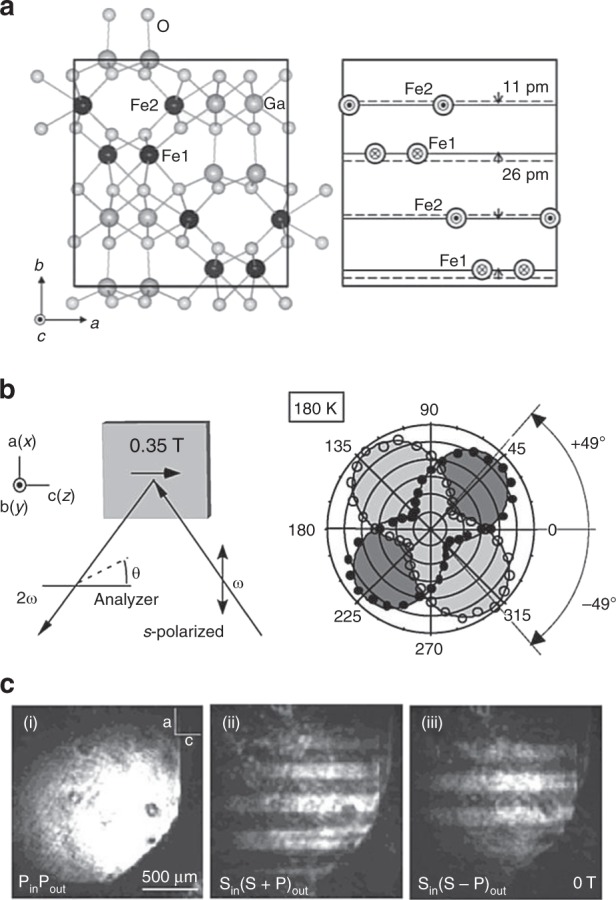
Second-harmonic (SH) generation from the toroidal moments in multiferroics. **a** Crystal and magnetic structures of GaFeO_3_. In the right panel, outward (inward) arrows represent the spin direction of Fe1 (Fe2) atoms antiparallel (parallel) to the *c* axis. Horizontal solid lines represent the shifted positions of Fe ions along the *b* axis from the symmetric positions (dashed lines) of FeO_6_ octahedra. Note that the shifted directions of Fe1 and Fe2 atoms are opposite to each other. Reprinted figure with permission from ref^60^. Copyright (2005) by the Physical Society of Japan. **b** Experimental configuration for the measurement of the Kerr rotation of SH light in a GaFeO_3_ crystal (left panel). Analyzer-angle dependence of SH intensity at 180 K (*<T*_*C*_). Analyzer angles *θ* of 0 and 90° stand for *p*- and *s*-polarized SH light, respectively. Solid and open circles indicate the SH intensity for +*z* and *–z* directions of the magnetic field, respectively. **c** (Left) topographic image of the *ac* surface of the GaFeO_3_ crystal as taken by the SH light in the nonmagnetic configuration, i.e., *P*in-*P*out. Magnetic domains aligned along the *c* axis were observed by reflected SH intensity in (center) *S*_in_(*S* + *P*)_out_ and (right) *S*_in_(*S–P*)_out_ configurations. Reprinted figure with permission from ref^59^. Copyright (2004) by the American Physical Society

Figure 5 exemplifies the toroidal moment-induced SHG in multiferroics^59^. FeGaO_3_ (Fig. 5a) shows the polar structure along the *b* axis (**P**//*b*), while the magnetic moments on two Fe sites (Fe1 and Fe2) is antiferromagnetically coupled with their moments parallel to the *c* axis. Then Fe1 and Fe2 jointly form the toroidal moment *T* //*a*^60^. The respective sublattice magnetization on Fe1 and Fe2 shows the unbalance, allowing the ferromagnetic moment *M*, which enables to control the sign of **T** //(**P** × **M**) by application of an external field. The SHG is originally active to produce **E**_2*ω*_//*b* light along the polar axis, while it is also activated by the toroidal moment for **E**_*ω*_//*a*. In the case of oblique incidence of the light with **E**_*ω*_//*a* (s-polarized) on the *ac* plane sample, both the SHG components (**E**_2*ω*_ //*b* from the original crystal polarity and **E**_2*ω*_ //*a* from the toroidal moment) can mix to generate the rotation of the polarization of the SHG light, as shown in Fig. 5b. This is called nonlinear Kerr rotation^61^ and the effect is usually much larger than the conventional Kerr rotation angle in ferromagnets. The reversal of either *P* or *M* can cause the sign reversal of the nonlinear Kerr rotation. The parallel component (say, *x*-component) of the oblique incident light ***k***-vector is either parallel or antiparallel to the applied magnetic field or magnetization direction (along *x*), and hence the reversal of *k*_*x*_ shows also the sign reversal of the nonlinear Kerr rotation. The phase (π) change of the *E*_2ω_ of the SH light upon the sign change of the toroidal moment enables one to image and map the toroidal moment domains in a multi-domain state, as exemplified in Fig. 5c. In some multiferroics, the ferrotoroidic/antiferrotoroidic domains can be distinguished from ferromagnetic/antiferromagnetic domains, as exemplified for the case of multiferroic LiCoPO_4_^62^. This method has been also applied to probe the magnetization at the heterointerface with the effective polarity along the normal^63,64^. It is also noted that the spin toroidization in periodic crystals has been formulated recently from the viewpoint of the Berry phase of Bloch wavefunctions^65^.

### Shift current in polar insulators

Up to now, we assume the broken time-reversal symmetry to obtain finite nonreciprocal response, e.g., the current proportional to *BE*^*2*^. However, one can ask if there is a possible mechanism of the current proportional *E*^*2*^ even without the broken $\hat T$-symmetry. From this viewpoint, it is known that the photoexcitation produces the current even without the potential gradient, i.e., the external electric field, in noncentrosymmetric materials. This photocurrent is called shift current. Figure **a** of Box 2 shows the experiment on a perovskite ferroelectrics [KNbO_3_]_1–x_[BaNi_1/2_Nb_1/2_O_3–δ_]_x_ (KBNNO), where the direction of the shift current is switched by that of the electric polarization^66^.

Then, an important question is how the nonreciprocal nonlinear responses are possible without the broken $\hat T$-symmetry? To answer to this question, one notes that the previous discussion was based on the energy dispersion satisfying the relation Eq. (7). Therefore, when the current does depend not only on the energy dispersion but also on the wavefunction, it is possible to realize the nonreciprocal responses without $\hat T$-breaking. This means that one must go beyond the Boltzmann transport theory where only the energy dispersion *ε*_*n*_(**k**) of the *n*th band and consequent group velocity appear in the equation. Namely, the group velocity **v**_*n*_(**k**) = ∂*ε*_*n*_(**k**)/∂**k** corresponds to the intraband matrix element of the current operator. On the other hand, the interband matrix elements of the current operator are related to the quantum geometry of the Bloch wavefunction and Berry phase^67^, playing essential roles in the various quantum transport phenomena including quantum Hall effect, quantum charge pumping, anomalous Hall effect, and spin Hall effect^68,69^. The Berry connection **a**_*n*_(**k**) is defined as **a**_*n*_(**k**) = *i*<*u*_*n*_(**k**)|∂/∂**k**|* u*_*n*_(**k**)> with |*u*_*n*_(**k**)> being the periodic part of the Bloch wavefunction. **a**_*n*_(**k**) is related to the overlap of the two Bloch wavefunctions neighboring in the momentum space as $< u_n\left( {\mathbf{k}} \right)|u_n\left( {{\mathbf{k}} + {\mathrm{\Delta }}{\mathbf{k}}} \right) > = e^{{\mathrm{i\Delta }}{\mathbf{k}} \cdot {\mathbf{a}}_n\left( {\mathbf{k}} \right)}$, and has the geometrical meaning of “connection” of manifold in Hilbert space. Therefore **a**_*n*_(**k**) is called Berry connection acting as the vector potential in the momentum space. From the vector potential, one can define the “magnetic field”, i.e., Berry curvature **b**_*n*_ (**k**) as **b**_*n*_(**k**) = ∇_**k**_ × **a**_*n*_(**k**). It is known that the Berry curvature causes the anomalous velocity of the electrons ${\mathbf{v}}_n^{{\mathrm{an}}}\left( {\mathbf{k}} \right) = {\mathbf{F}} \times {\mathbf{b}}_n\left( {\mathbf{k}} \right)$ under the force **F** acting on the electrons. This anomalous velocity is regarded as the origin of the intrinsic anomalous Hall effect in ferromagnets^69^. When the symmetries $\hat I$ and $\hat T$ are valid, there occurs the Kramer’s doublet at each *k*-point, and therefore the Berry phase should be defined as the 2 × 2 matrix, i.e., SU(2), instead of the U(1) phase as discussed before. In other words, the U(1) Berry curvature is zero in this case. On the other hand, when $\hat I$*-*symmetry is broken, the Kramer’s degeneracy is lifted at each *k*-point in the presence of the spin–orbit interaction, and U(1) Berry phase can be nonzero. Therefore, the noncentrosymmetry is encoded in the U(1) Berry phase. (Note that $\hat T$-breaking also produces the Berry phase in the presence of the spin–orbit interaction.)

Since the real-space position *r* of the wavepacket made from the Bloch wavefunctions is given by the gauge covariant derivative as **r**_*n*_(**k**) = *i*∇_**k**_ + **a**_*n*_(**k**), **a**_*n*_ (**k**) has the meaning of intracell coordinate^70^. The interband transition from *n*-band to *m*-band induced by the incident light results in the change of this intracell coordinate from **a**_*n*_ (**k**) to **a**_*m*_ (**k**) and the corresponding shift of the electron by the amount of **r**_*nm*_(**k**) = ∇_**k**_*φ*_*nm*_(**k**) + **a**_*n*_(**k**)–**a**_*m*_(**k**) (shift vector) where the first term makes the **r**_*nm*_ gauge invariant with *φ*_*nm*_(**k**) being the phase of the interband matrix element of the current operator. Therefore, in the steady state under the light irradiation, the continuous shift of the electrons by **r**_*nm*_ gives the dc current called shift current^66,71–75^. This shift current has been estimated as a function of incident light energy for BaTiO_3_ by the first-principles calculation, and a good agreement was obtained between theory and experiment^73^.

Shift current is essentially different from the conventional transport current in the sense that the former comes from the interband matrix elements of the current *J*, while the latter from the intraband ones^71–75^. Therefore, it is analogous to the polarization current in ferroelectrics, but the remarkable feature is that it can be the dc current. The experimental results on tetrathiafulvalene-*p*-chloranil (TTF-CA) which is a quasi-one-dimensional compound consisting of mixed stacks of alternating donor (TTF) and acceptor (CA) molecules are shown in Box 2 ^76^. Shift current is a rather ubiquitous phenomenon in noncentrosymmetric systems. Actually, shift current has been studied or discussed for GaAs^77^, SbSI^78^, and warped surface state of the three-dimensional TI^79^. Furthermore, the shift spin current can be also considered, which can have an application to spintronics^79^.

### Box 2

Shift current has been proposed theoretically^71,72^, and recently attracts intensive interest as a possible mechanism of the highly efficient solar cell action in noncentrosymmetric crystals such as perovskite oxides. Figure **a** shows a representative example, i.e., ferroelectric oxide [KNbO_3_]_1–*x*_[BaNi_1/2_Nb_1/2_O_3*–δ*_]_*x*_ (KBNNO)^66^. This materials shows the ferroelectric behavior, and the spontaneous electric polarization can be switched by an external electric field. Correspondingly, the direction of the photocurrent changes as shown in Fig. **b**. Figures **c**–**e** show another example, i.e., charge transfer organic semiconductor tetrathiafulvalene-p-chloranil (TTF-CA), where the shift current is considered to be the main mechanism of photovoltaic effect^76^. Figure **c** shows its crystal structure made of alternating donor and acceptor molecules, and Fig. **d** the temperature dependence of the photocurrent without the applied electric field (short-circuit current). It is seen that the photocurrent grows rapidly below *T*c. Figure **e** shows the light irradiation position dependence of the photocurrent at above and below *T*c. Above *T*c, it shows the maximum near the electrodes and the sign is the opposite between the right and left. This can be interpreted naturally that the photoinduced electrons and holes diffuse and are easily captured by the electrodes near the ends of the sample. Below *T*c, this behavior changes dramatically as well as the magnitude of the photocurrent. It is almost independent of the local position of light irradiation, which indicates the nonlocal nature of the shift current. A recent theoretical study reproduces this behavior on the basis of the numerical simulation of Rice–Mele model under the local light irradiation^89^. **Shift current in polar perovskite system and charge transfer organic compound.** **a** Crystal structure of the ferroelectric oxide [KNbO_3_]_1*–x*_[BaNi_1/2_Nb_1/2_O_3–*δ*_]_*x*_ (KBNNO) used in DFT band structure calculation. K and Ba are shown by blue and green spheres, respectively. **b** The time-dependence of the photocurrent induced by the pulse excitation without the applied electric field. Red and blue curves correspond to the opposite directions of the poling electric field and electric polarization. The inset shows the applied voltage dependence of the photocurrent. Figure from ref.^66^ with permission from Nature Publishing Group. **c** Molecular structures of TTF (donor) and CA (acceptor) (left), and the crystal structure of TTF-CA, which consists of alternating donor and acceptor molecules along *a*-axis. This material shows a ferroelectric transition at *T*_C_ = 81 K due to the dimerization. **d** The temperature dependence of the shift current in TTF-CA, which becomes finite below *T*_C_. The inset shows the temperature dependence of the electric polarization. **e** The dependence of the photocurrent on the position of the local optical excitation. Above *T*_C_ (blue), it shows maximum near the electrodes with the opposite signs, and nearly zero at the center of the sample. This is consistent with the diffusion model of photocarriers created by the optical excitation. Below *T*_C_, the magnitude of the photocurrent is enhanced and also shows the almost constant behavior in the middle region the sample in sharp contrast to that above *T*_C_. This is interpreted as the onset of the shift current below *T*_C_^76^

### Giant second-harmonic generation in Weyl semimetals

**Fig. 6:**
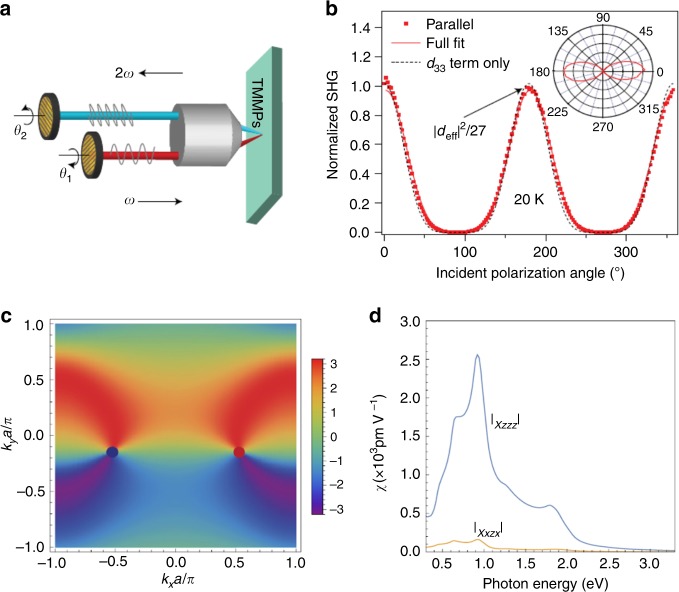
Second-harmonic generation (SHG) in TaAs. **a** Schematic of the SHG experimental setup. **b** SHG intensity as a function of angle of incident polarization at 20 K. **c** Color plot of the momentum-resolved contribution to the SHG for a model for a simplified mode for TaAs. **d** The photon-energy dependence of SHG for a simplified model. Figures from ref^80^. Reprinted with persmission from Nature Publishing Group

The shift current is the dc current induced by the optical excitation, and is the second-order process where the current is proportional to *E*(*ω*)*E*(−*ω*). Similarly, the ac current with frequency 2*ω* proportional to *E*(*ω*)*E*(*ω*) can be produced, i.e., second-harmonic generation (SHG). It has been discussed that SHG has the similar expression in terms of the shift vector **r**_*nm*_(**k**) = ∇_**k**_φ_*nm*_(**k**)+**a**_*n*_(**k**)–**a**_*m*_(**k**) defined above, and again has the geometrical meaning. Especially, the Weyl point corresponds to the magnetic (anti)monopole of the Berry connection^49^, and hence can be a source of large SHG. Actually as shown in Fig. 6, in noncentrosymmetric Weyl semimetal TaAs, the large SHG has been observed with the magnitude of |*d*|≈3600 pmV^−1^ (*d* represents a component of the third-rank tensor representing SHG). This large value is to be compared with corresponding values in other materials such as |*d*|≈350 pmV^−1^ for GaAs, |*d*|≈250 pmV^−1^ for ZnTe, and |*d*|≈15 pmV^−1^ for BaTiO_3_^80^. SHG of a model with two Weyl fermions has been analyzed, and reasonably consistent values with the experiment were obtained.

### Nonlinear photocurrent induced by Berry curvature

The photocurrent discussed above is driven by the Berry connection **a**_*n*_(**k**) rather than the Berry curvature **b**_*n*_(**k**). Then it is natural to ask if there is a photocurrent induced by **b**_*n*_(**k**). This question has been studied by Moore and Orenstein, who predicted the helicity-dependent photocurrent due to Berry curvature^81^. The idea is that the deviation *δf*_*n*_(*k*,*t*) of the electron distribution function occurs in the presence of the electric field *E* and is proportional to τ(∂*f*(ε)/∂ε)*v*_*n*_(**k**)*E* with being the relaxation time and *v*_*n*_(**k**) the group velocity. The integral of the anomalous velocity ${\mathbf{v}}_n^{{\mathrm{an}}}\left( {\mathbf{k}} \right) = - e{\mathbf{E}} \times {\mathbf{b}}_n\left( {\mathbf{k}} \right)$ over the modified distribution function results in the photocurrent proportional to *E*^2^. The photocurrent is induced by this mechanism for the quantum wells structure with the quantum confinement of electrons^81^. A similar idea has also been applied to the nonlinear Hall effect in time-reversal symmetric systems induced by Berry curvature dipole^82^.

### Perspective

In this article, we have seen ample examples of nonreciprocal responses from quantum materials with broken space-inversion symmetry $\hat I$. The directional transport/propagation of the quanta, such as electron, photon, spin, and phonon, enables the one-way transmission of the information carrier. As shown in Box 1, the diagonal linear nonreciprocal response is only allowed for the system with broken time-reversal symmetry, namely in a magnetic field or in a magnetically ordered state such as the multiferroics. As the linear response, the nonreciprocal transport of magnon can substantiate the diode effect of spin current, which may append an important spintronic function. The directional dichroism response is also an important nonreciprocal function in the multiferroics that can support the ME excitations, typically electromagnons. The directional dichroism can realize the cloaking function; contrary to the conventional Faraday rotation, it does not need the additional analyzer of the light polarization to achieve the one-way transmission of light, if the effect can be tuned so as to nearly equalize the electric-dipole and magnetic-dipole transition moments.

Let us turn our eyes to the nonreciprocal transport of an electron. The p–n junction (diode) is the most successful and spectacular example for this. Although we did not touch on this classic example in this article, it originates from the change in the width of the depletion layers at the junction, and can be attributed to the Coulombic interaction of electrons, i.e., the electron correlation effect. Recently, this issue has been revisited from the viewpoint of the multiband effect^83^. As for the dc electric field, only the inclusion of the electron–electron interaction can produce the nonreciprocal current proportional to *E*^2^, while the non-interacting electron system cannot.

The shift current is another example that does not need breaking of time-reversal symmetry, but even the electron correlation is not required in this case. Its topological nature may ensure the ballistic photocurrent upon the photoexcitation. In addition to its importance as the initial process of the photovoltaic action, the shift current may also work as potentially ultrafast information transfer. As exemplified by the p–n junction and the shift current, the nonlinear nonreciprocity is the key to realize the ac-to-dc conversion. On this basis, the dc spin current generation is also possible by the ac (photo) excitation on the surface state of TI^79^. In the magnetic system with broken time-reversal symmetry $\hat T$, an even more variety of the nonlinear and nonreciprocal current controls are possible, as described in this article for the nonlinear conduction for the Rashba system under a magnetic field, the semi-magnetic TI, and the chiral-lattice magnet. There, one mechanism of unidirectional magnetoresistance is the field (magnetization)-induced electronic structural change even with the constant scattering time as in the Rashba system, and the other is due to the carrier scattering itself, for example, the backward scattering via magnon emission/absorption in a TI and the directional scattering due to chiral spin fluctuation in the chiral-lattice magnet (MnSi). Thus, the study on the nonlinear and nonreciprocal charge transport can bring about the important information about the underlying charge dynamics in the simultaneously broken symmetries of time-reversal and space-inversion.

One interesting question is whether the nonreciprocal responses are possible when the product of the symmetries $\hat I\hat T$ is intact while both $\hat I$ and $\hat T$ are broken. One can easily see that this product symmetry does not exclude the linear nonreciprocal responses. For example, the toroidal moment, which produces the directional dichroism, breaks both $\hat I$ and $\hat T$ symmetries, while it does not break $\hat I\hat T.$. Of particular interest is the $\hat I\hat T$symmetry in non-Hermitian systems. (Usually it is called *PT*-symmetry^84^.) It has been found that *PT*-symmetry plays a crucial role in enhancing the optical isolator function^85,86^. In addition to optics, the nonreciprocal propagation of heat is also a hot topic^87^. Also the relation to the quantum Ratchet is an interesting problem to be explored more in depth^88^. In this case, there is no $\hat T$ symmetry breaking and the soliton-like nonlinear excitations are regarded as the source of the thermal rectification effect.

From the viewpoint of the basic principles, the noncentrosymmetry is encoded by the Berry phase, and the Bloch wavefunctions acquire the quantum geometric properties as discussed in section “Nonlinear/nonreciprocal photonic responses.” Berry phase is related to the interband matrix elements of the current operator, and produces new types of current in sharp contrast to the conventional transport current due to the intraband matrix elements which allows the particle picture through the wavepacket formalism. This “interband current” manifests itself as the shift current appearing as the photovoltaic effect. Furthermore, it should be noted that the electron correlation combined with the interband current matrix elements plays an essential role in the nonreciprocal responses. Therefore, the physics of nonreciprocal responses touch the most important elements of modern condensed matter physics, i.e., the symmetry, quantum geometry or topology, electron correlation, and also irreversibility. Reducing the symmetries in space and/or time in quantum materials have explored a fertile ground for condensed matter physics and electronics. The recent research of multiferroics exemplifies this. Exploring the nonreciprocal responses in quantum and topological materials as described here is a promising direction of the research in search for emergent functions.

## References

[1] Gardner M (1964). The Ambidextrous Universe. Left, Right and the Fall of Parity.

[2] Onsager L (1931). Reciprocal relations in irreversible processes. I. Phys. Rev..

[3] Landau, L. D.; Lifshitz, E. M. (1975). *Statistical Physics, Part 1*. Oxford, UK. Butterworth-Heinemann. ISBN 978-981-8147-790-3.

[4] Kubo R (1957). Statistical-mechanical theory of irreversible processes. I. General theory and simple applications to magnetic and conduction problems. J. Phys. Soc. Jpn..

[5] Rikken GLJA, Raupach E (1997). Observation of magneto-chiral dichroism. Nature.

[6] Rikken GLJA, Strohm C, Wyder P (2002). Observation of magnetoelectric directional anisotropy. Phys. Rev. Lett..

[7] Shimada Y, Kiyama H, Tokura Y (2006). Magnetoelectric emissio in magnetic ferroelectric Er-doped (Ba, St)TiO_3_. Appl. Phys. Lett..

[8] Note that magnetoelectric Jones birefringence with E ∙ B term has been reported in Roth, T. & Rikken, G.L.J.A. Observation of magnetoelectric Jones birefringence. *Phys. Rev. Lett*. **85**, 4478-4481 (2000). 10.1103/PhysRevLett.85.4478 11082575

[9] Tokura Y, Seki S, Nagaosa N (2014). Multiferroics of spin origin. Rep. Prog. Phys..

[10] Seki S (2007). Impurity-doping-induced ferroelectricity in the frustrated antiferromagnet CuFeO_2_. Phys. Rev. B.

[11] Arima T (2007). Ferroelectricity induced by proper-screw type magnetic order. J. Phys. Soc. Jpn..

[12] Kibayashi S, Takahashi Y, Seki S, Tokua Y (2014). Magnetochiral dichroism resonant with electromagnons in a helimagnet. Nat. Commun..

[13] Spaldin NA, Fechner M, Bousquet E, Balatsky A, Nordstrom L (2013). Monopole-based formalism for the diagonal magnetoelectric response. Phys. Rev. B.

[14] Dzyaloshinskii IE (1960). On the magneto-electrical effect in antiferromagnets. JETP.

[15] Astrov DN (1960). The magnetoelectric effect in antiferromanets. Sov. Phys. JETP-USSR.

[16] Krichetsov BB, Pavlov VV, Pisarev RV, Gridnev VN (1993). Spontaneous nonreciprocal reflection of light from antiferromagnetic Cr_2_O_3_. J. Phys. -Cond. Mat..

[17] Kurumaji T, Ishiwata S, Tokura Y (2015). Doping-tunable ferromagnetic phase with large linear magnetoelectric effect in a polar magnet Fe_2_Mo_3_O_8_. Phys. Rev. X.

[18] Kurumaji T (2017). Optical magnetoelectric resonance in a polar magnet (Fe, Zn)_2_Mo_3_O_8_ with axion-type coupling. Phys. Rev. Lett..

[19] Qi XL, Hughes TL, Zhang SC (2008). Topological field theory of time-reversal invariant insulators. Phys. Rev. B.

[20] Morimoto T, Furusaki A, Nagaosa N (2015). Topological magnetoelectric effects in thin films of topological insulators. Phys. Rev. B.

[21] Mogi M (2017). A magnetic heterostructure of topological insulators as a candidate for an axion insulator. Nat. Mater..

[22] Okamura Y (2015). Microwave magnetochiral dichroism in the chiral-lattice magnet Cu_2_OSeO_3_. Phys. Rev. Lett..

[23] Kubota M (2004). X-ray directional dichroism of a polar ferrimagnet. Phys. Rev. Lett..

[24] Takahashi Y, Shimano R, Kaneko Y, Murakawa H, Tokura Y (2012). Magnetoelectric resonance with electromagnons in a perovskite helimagnet. Nat. Phys..

[25] Kezsmarki I (2011). Enhanced directional dichroism of terahertz light in resonance with magnetic excitations of the multiferroic Ba2CoGe2O7 oxide compound. Phys. Rev. Lett..

[26] Toyoda S (2015). One-way transparency of light in multiferroic Cu_B2O4_. Phys. Rev. Lett..

[27] Kezsmarki I (2014). One-way transparency of four-coloured spin-wave excitations in multiferroic materials. Nat. Commun..

[28] Iguchi Y, Uemura S, Ueno K, Onose Y (2015). Nonreciprocal magnon propagation in a noncentrosymmetric ferromagnet LiFe5O8. Phys. Rev. B.

[29] Seki S (2016). Magnetochiral nonreciprocity of volume spin wave propagation in chiral-lattice ferromagnets. Phys. Rev. B.

[30] Sato TJ (2016). Magnon dispersion shift in the induced ferromagnetic phase of noncentrosymmetric MnSi. Phys. Rev. B.

[31] Takagi R (2017). Spin-wave spectroscopy of the Dzyalonshinskii-Moriya interaction in room-temperature chiral magnets hosting skyrmions. Phys. Rev. B.

[32] Cho J (2015). Thickness dependence of the interfacial Dzyaloshinskii-Moriya interaction in inversion symmetry broken systems. Nat. Commun..

[33] Membach HT, Shaw JM, Weiler M, Jue E, Silva TJ (2015). Linear relation between Heisenberg exchange and interfacial Dzyaloshinskii-Moriya interaction in metal films. Nat. Phys..

[34] Evans DJ, Cohen EDG, Morris GP (1993). Probability of second law violations in shearing steady states. Phys. Rev. Lett..

[35] Kurchan J (1998). Fluctuation theorem for stochastic dynamics. J. Phys. A (Math. Gen.).

[36] Jarzynski C (2000). Hamiltonian derivation of a detailed fluctuation theorem. J. Stat. Phys..

[37] Esposito M, Harbola U, Mukamel S (2014). Nonequilibrium fluctuations, fluctuations theorems, and counting statistics in quantum systems. Rev. Mod. Phys..

[38] Saito K, Utsumi Y (2008). Symmetry in full counting statistics, fluctuation theorem, and relations among nonlinear transport coefficients in the presence of a magnetic field. Phys. Rev. B.

[39] Nakamura S (2011). Fluctuation theorem and microreversibility in a quantum coherent conductor. Phys. Rev. B.

[40] Rikken GLJA, Folling J, Wyder P (2001). Electrical magnetochiral anisotropy. Phys. Rev. Lett..

[41] Rikken GLJA, Wyder P (2005). Magnetoelectric anisotropy in diffusive transport. Phys. Rev. Lett..

[42] Avci CO (2015). Unidirectional spin Hall magnetoresistance in ferromagnet/normal metal bilayers. Nat. Phys..

[43] Ideue T (2017). Bulk rectification effect in a polar semiconductor. Nat. Phys..

[44] Ishizaka K (2011). Giant Rashba-type spin splitting in bulk BiTeI. Nat. Mater..

[45] Yasuda K (2016). Large unidirectional magnetoresistance in a magnetic topological insulator. Phys. Rev. Lett..

[46] Krstić V, Roth S, Burghard M, Kern K, Rikken GLJA (2002). Magneto-chiral anisotropy in charge transport through single-walled carbon nanotubes. J. Chem. Phys..

[47] Pop F, Auban-Senzier P, Canadell E, Rikken GLJA, Avarvari N (2014). Electrical magnetochiral anisotropy in a bulk chiral molecular conductor. Nat. Commun..

[48] Yokouchi T (2017). Electrical magnetochiral effect induced by chiral spin fluctuations. Nat. Commun..

[49] Vafek O, Vishwanath A (2014). Dirac fermions in solids: from high-T-c cuprates and graphene to topological insulators and Weyl semimetals. Annu. Rev. Condens. Matter Phys..

[50] Nielsen HB, Ninomiya M (1981). *A no-go theorem for regularizing chiral fermions*. Phys. Lett. B.

[51] Li Qiang (2016). Chiral magnetic effect in ZrTe_5_. Nat. Phys..

[52] Morimoto T, Nagaosa N (2016). Chiral anomaly and giant magnetochiral anisotropy in noncentrosymmetric Weyl semimetals. Phys. Rev. Lett..

[53] For a review of noncentrosymmetric superconductors, see Bauer, E., Sigrist, M. Eds., *Non-Centrosymmetric Superconductors*, (Springer Berlin Heidelberg, Heidelberg, 2012).

[54] Saito Y, Nojima T, Iwasa Y (2017). Highly crystalline 2D superconductors. Nat. Rev. Mater..

[55] Ye JT (2012). Superconducting dome in a gate-tuned band insulator. Science.

[56] Wakatsuki (2017). Nonreciprocal charge transport in noncentrosymmetric superconductors. Sci. Adv..

[57] Schmid A (1969). Diamagnetic susceptibility at the transition to the superconducting state. Phys. Rev..

[58] Spaldin NA, Fiebig M, Mostovoy M (2008). The toroidal moment in condensed-matter physics and its application to the magnetoelectric effect. J. Phys. Condens. Matter.

[59] Ogawa Y (2004). Magnetization-induced second harmonic generation in a polar ferromagnet. Phys. Rev. Lett..

[60] Arima T (2005). Resonant magnetoelectric X-ray scattering in GaFeO_3_: observation of ordering of toroidal moments. J. Phys. Soc. Jpn..

[61] Koopmans B, Koerkamp MG, Rasing T, Vandenberg H (1995). Observation of large Kerr angles in the nonlinear-optical response from magnetic multilayers. Phys. Rev. Lett..

[62] Van Aken BB, Rivera JP, Schmid H, Fiebig M (2007). Observation of ferrotoroidic domains. Nature.

[63] Yamada H (2004). Engineered interface of magnetic oxides. Science.

[64] Hwang HY (2012). Emergent phenomena at oxide interfaces. Nat. Mater..

[65] Gao Y, Vanderbilt D, Xiao D (2018). Microscopic theory of spin toroidization in periodic crystals. Phys. Rev. B.

[66] Grinberg I (2013). Perovskite oxides for visible-light-absorbing ferroelectric and photovoltaic materials. Nature.

[67] Berry MV (1984). Quantal phase factors accompanying adiabatic changes. Proc. R. Soc. Lond..

[68] Xiao D, Chang MC, Niu Q (2010). Berry phase effects on electronic properties. Rev. Mod. Phys..

[69] Nagaosa N, Sinova J, Onoda S, MacDonald AH, Ong NP (2010). Anomalous Hall effect. Rev. Mod. Phys..

[70] Adams EN, Blount EI (1959). Energy bands in the presence of an external force field–II. Anomalous velocities. J. Phys. Chem. Solids.

[71] Vonbaltz R, Kraut W (1981). Theory of the bulk photo-voltaic effect in pure-crystals. Phys. Rev. B.

[72] J. E. Sipe JE, Shkrebtii AI (2000). Second-order optical response in semiconductors. Phys. Rev. B.

[73] Young SteveM, Zheng Fan, Rappe AndrewM (2012). First-principles calculation of the bulk photovoltaic effect in bismuth ferrite. Phys. Rev. Lett..

[74] Morimoto T, Nagaosa N (2016). Topological nature of nonlinear optical effects in solids. Sci. Adv..

[75] Nagaosa N, Morimoto T (2017). Concept of quantum geometry in optoelectronic processes in solids: application to solar cells. Adv. Mater..

[76] Nakamura M (2017). Shift current photovoltaic effect in a ferroelectric charge-transfer complex. Nat. Commun..

[77] Cote D, Laman N, van Driel HM (2002). Rectification and shift current in GaAs. Appl. Phys. Lett..

[78] Ogawa N, Sotome M, Kankeo Y, Ogino M, Tokura Y (2017). Shift current in the ferroelectric semiconductor SbSI. Phys. Rev. B.

[79] Kim KS, Morimoto T, Nagaosa N (2017). Shift charge and spin photocurrents in Dirac surface states of topological insulator. Phys. Rev. B.

[80] Wu L (2017). Giant anisotropic nonlinear optical response in transition metal monopnictide Weyl semimetals. Nat. Phys..

[81] Moore JE, Orenstein J (2010). Confinement-induced Berry phase and helicity-dependent photocurrents. Phys. Rev. Lett..

[82] Sodemann I, Fu L (2015). Quantum nonlinear Hall effect induced by Berry curvature dipole in time-reversal invariant materials. Phys. Rev. Lett..

[83] Morimoto T, Nagaosa N (2018). Nonreciprocal current from electron interactions in noncentrosymmetric crystals: roles of time reversal symmetry and dissipation. Sci. Rep..

[84] Bender CM, Boettcher S (1998). Real spectra in non-Hermitian Hamiltonians having PT symmetry. Phys. Rev. Lett..

[85] Bender N (2013). Observation of asymmetric transport in structures with active nonlinearities. Phys. Rev. Lett..

[86] Cang L (2014). Parity–time symmetry and variable optical isolation in active– passive-coupled microresonators. Nat. Photonics.

[87] C. W. Chang CW, Okawa D, Majumdar A, Zettl A (2006). Solid-state thermal rectifier. Science.

[88] Reimann Peter (2002). Brownian motors: noisy transport far from equilibrium Peter Reimann. Phys. Rep..

[89] Ishizuka H, Nagaosa N (2017). Local photo-excitation of shift current in noncentrosymmetric systems. New J. Phys..

